# Supplementary material for: Flexing with lines or pipes: Techno-economic comparison of renewable electricity import options for European research facilities
Source: PLoS One. 2024 Feb 8;19(2):e0292892. doi: 10.1371/journal.pone.0292892 (PMC10852270; doi:10.1371/journal.pone.0292892)
Supplement: S1 Appendix — (PDF) [file pone.0292892.s001.pdf]

## S1 Appendix. Tabular results of Levelised Cost of Electricity

**Table A. Tabular results of LCoEs in EUR/MWh for all scenarios modeled for this study.**
Values are visualized in Fig 3.

| | CSP | with CSP | | | | no CSP | | | |
|---|---|---|---|---|---|---|---|---|---|
| | ESC | HVDC | | $H_2$ pipeline | | HVDC | | $H_2$ pipeline | |
| | Exporter | MA | TN | MA | TN | MA | TN | MA | TN |
| Flexibility | | | | | | | | | |
| Annual | | 59 | 63 | 157 | 159 | 71 | 68 | 164 | 163 |
| Quarterly | | 59 | 63 | 157 | 159 | 71 | 68 | 164 | 166 |
| Monthly | | 59 | 63 | 157 | 159 | 71 | 68 | 164 | 167 |
| Biweekly | | 59 | 63 | 157 | 159 | 72 | 75 | 164 | 169 |
| Weekly | | 60 | 67 | 160 | 168 | 77 | 82 | 167 | 174 |
| Daily | | 65 | 76 | 172 | 190 | 83 | 95 | 180 | 195 |
| Baseload | | 71 | 82 | 178 | 196 | 101 | 107 | 187 | 201 |
