# Supplementary material for: Flexing with lines or pipes: Techno-economic comparison of renewable electricity import options for European research facilities
Source: PLoS One. 2024 Feb 8;19(2):e0292892. doi: 10.1371/journal.pone.0292892 (PMC10852270; doi:10.1371/journal.pone.0292892)
Supplement: S2 Appendix — (PDF) [file pone.0292892.s002.pdf]

S2 Appendix. Technology assumption details

Table B. Details on the technology assumptions made in the model for 2030, as listed in Table 1.
The *currency year* indicates the monetary year of all costs taken from the specific publication. Where the *currency year* is not 2015, inflation adjustment is done assuming 2 % p.a. inflation rate.

**Technology**	Source	Currency year	Additional information
Wind onshore	[2]	2015	Technology "20 Onshore turbines".
Wind offshore	[2]	2020	Technology "21 Offshore turbines" nominal investment minus grid connection costs.
Solar PV	[2]	2020	Technology "22 Utility-scale PV".
CSP field & receiver tower	[1], [3]	2020	CAPEX based on [1] 2020 numbers, combined with CAPEX degression taken from ATB database "moderate" scenario; costs include solar field, solar tower, and EPC cost for the default installation size of 104 MW(e) plant. Total costs (223,708,924 USD) are divided by active area (heliostat reflective area, 1,269,054 m2) and multiplied by design point DNI (0.95 kW/m2) to obtain EUR per kW_{th} value. Exchange rate: 1.16 USD to 1 EUR. FOM calculated from the ratio between CAPEX and FOM of [3] for CSP in "Moderate" scenario. Lifetime taken from [3].
CSP TES	[1], [3]	2020	CAPEX based on [1] 2020 numbers, combined with CAPEX degression taken from ATB database "moderate" scenario; costs include the TES and EPC cost for the default installation size 104 MW(e) plant and 2791 MW(th) TES. Total costs (69390776.7 USD) are divided by TES size to obtain EUR per kW(th). Exchange rate: 1.16 USD to 1 EUR. FOM calculated from the ratio between CAPEX and FOM of [3] for CSP in "Moderate" scenario. Lifetime taken from [3].
CSP power block	[1], [3]	2020	CAPEX based on [1] 2020 numbers, combined with CAPEX degression taken from ATB database "moderate" scenario; costs include the power cycle incl. BOP and EPC cost for the default installation size (104 MWe plant). Total costs (135185685.5 USD) are divided by power block nameplate capacity size to obtain EUR per kW(el). Exchange rate: 1.16 USD to 1 EUR. FOM calculated from the ratio between CAPEX and FOM of [3] for CSP in "Moderate" scenario. Lifetime taken from [3].
Battery storage	[4]	2015	Technology "180 Lithium Ion Battery", Energy storage expansion cost investment.
Battery inverter	[4]	2015	Technology "180 Lithium Ion Battery", Output capacity expansion cost investment.
Electrolysis (Alkaline)	[5]	2015	Technology "86 AEC 100MW".

Continue on the next page

Table B (cont.)

**Technology**	Source	Currency year	Additional information
Hydrogen storage tank	[6]	2020	Table SI.9, technology “GH2 (L)” with 450 EUR per kg of h2 converted to MWh using LHV with LHV to MWh. The currency year assumed 2020 for the initial publication of reference and observing note in SI.4.3 that in their reference no currency year is explicitly stated.
HVDC inverter pair	[7]	2011	Table A.2
HVDC line overhead on-land	[7]	2011	Table A.2
HVDC line underwater	[8]	2018	Estimated costs for a connector between Europe and North America (bidirectional, 4 GW, ca. 3000 km length and 3000m depth). Costs from publication are based on existing and currently under construction undersea cables.
H_2 (g) pipeline fill station	[9], [10]	2015	CAPEX from [9] from figure 14 of PDF, pg. 164, for staging 35 to 140 bar at a capacity of 6000 MW(HHV) single line pipeline and converting for LHV of H2. Lifetime from [9] figure 24 of PDF, pg. 168. FOM from [10] table 3 and table 5 with the pessimistic (highest) value chosen for a 48-inch pipeline with 13 GW_{LHV} at 100 bar pressure; the forecast year is not specified, assumed 2020 based on remarks in PDF.
H_2 (g) pipeline on-land	[11], [9]	2020	CAPEX Assumption for a 48-inch single line pipeline, incl. compressor investments, 16.9 GW peak capacity, 2750 EUR/m, 434 MWe/1000 km for compressor, 3.4 MEUR/MWe for compressor, from [11] table 35. FOM and lifetime from [9] Assumption for a 140 bar, $>$ 6000 MW HHV single line pipeline, incl. booster station investments. Considering LHV by scaling with LHV/HHV=0.8462623413.
H_2 (g) pipeline underwater	[12], [13]	2014	CAPEX oriented on to [12] for 36-inch CH4 submarine pipeline multiplied by 2.86 which is a common factor found for cost estimates between CH4 and H2 pipelines. FOM and lifetime from [13], assuming the same as for a CH4 pipeline.
H_2 gas turbine (CCGT)	[2]	2015	Technology “05 Gas turb. CC, steam extract.”.
Demand-side flexibility	-	-	Guesstimate.

References

1. National Renewable Energy Laboratory. System Advisor Model Version 2021.12.02 (SAM 2021.12.02); 2021. Available from: https://sam.nrel.gov.
2. Danish Energy Agency, Energinet. Technology Data for Generation of Electricity and District Heating (Version 11, 03/2022); 2022. Available from: https://ens.dk/sites/ens.dk/files/Analyser/version_11_-_technology_data_for_el_and_dh.xlsx.
3. National Renewable Energy Laboratory. 2021 Annual Technology Baseline; 2021. Available from: https://atb.nrel.gov/.
4. Danish Energy Agency, Energinet. Technology Data Catalogue for Energy Storage - October 2018 - Updated January 2020; 2020. Available from: https://ens.dk/en/our-services/projections-and-models/technology-data/technology-data-energy-storage.
5. Danish Energy Agency, Energinet. Technology Data for Renewable Fuels Version 8 (04/2021); 2021. Available from: https://ens.dk/sites/ens.dk/files/Analyser/technology_data_for_renewable_fuels.pdf.
6. Stöckl F, Schill WP, Zerrahn A. Optimal Supply Chains and Power Sector Benefits of Green Hydrogen. Scientific Reports. 2021;11(1):14191. doi:10.1038/s41598-021-92511-6.
7. Hagspiel S, Jägemann C, Lindenberger D, Brown T, Cherevatskiy S, Tröster E. Cost-Optimal Power System Extension under Flow-Based Market Coupling. Energy. 2014;66:654–666. doi:10.1016/j.energy.2014.01.025.
8. Purvins A, Sereno L, Ardelean M, Covrig CF, Efthimiadis T, Minnebo P. Submarine Power Cable between Europe and North America: A Techno-Economic Analysis. Journal of Cleaner Production. 2018;186:131–145. doi:10.1016/j.jclepro.2018.03.095.
9. Danish Energy Agency, Energinet. Technology Data for Energy Transport Version 03 (03/2021); 2021. Available from: https://ens.dk/en/our-services/projections-and-models/technology-data/technology-catalogue-transport-energy.
10. Anthony Wang, Kees van der Leun, Daan Peters, Maud Buseman. European Hydrogen Backbone: How a Dedicated Hydrogen Infrastructure Can Be Created; 2020. Available from: https://guidehouse.com/insights/energy/2020/developing-europes-hydrogen-infrastructure-plan.
11. Anthony Wang, Jaro Jens, David Mavins, Marissa Moultak, Matthias Schimmel, Kees van der Leun, et al. European Hydrogen Backbone: Analysing Future Demand, Supply, and Transport of Hydrogen; 2021. Available from: https://gasforclimate2050.eu/wp-content/uploads/2021/06/EHB_Analysing-the-future-demand-supply-and-transport-of-hydrogen_June-2021.pdf.
12. Kaiser MJ. Offshore Pipeline Construction Cost in the U.S. Gulf of Mexico. Marine Policy. 2017;82:147–166. doi:10.1016/j.marpol.2017.05.003.
13. d'Amore-Domenech R, Leo TJ, Pollet BG. Bulk Power Transmission at Sea: Life Cycle Cost Comparison of Electricity and Hydrogen as Energy Vectors. Applied Energy. 2021;288:116625. doi:10.1016/j.apenergy.2021.116625.
