# Supplementary material for: Flexing with lines or pipes: Techno-economic comparison of renewable electricity import options for European research facilities
Source: PLoS One. 2024 Feb 8;19(2):e0292892. doi: 10.1371/journal.pone.0292892 (PMC10852270; doi:10.1371/journal.pone.0292892)
Supplement: S3 Appendix — (PDF) [file pone.0292892.s003.pdf]

S3 Appendix. Extended and additional figures

S3A Appendix. Import cost compositions

In Fig 7 the cost compositions for ESCs from MA with CSP are shown. An extended version of this figure is shown below in Fig A for all flexibility scenarios, scenarios without CSP, and all scenarios from TN.

The categories used in Fig 7 and the figures below are the following:

- RES: Electricity generation from offshore wind, onshore wind, PV and CSP; excluding TES
- Storage: Battery storage, TES, H_2 storage
- Transport: All HVDC and H_2 pipeline components, incl. inverter and compressor stations
- H2 electrolysis: H_2 electrolysis for converting water and electricity to H_2
- H2 gas turbine: CCGT cost for converting H_2 back to electricity

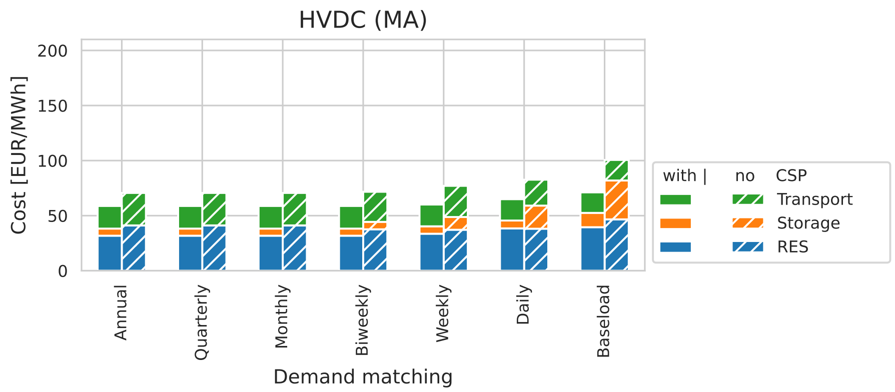

(a) HVDC from MA.

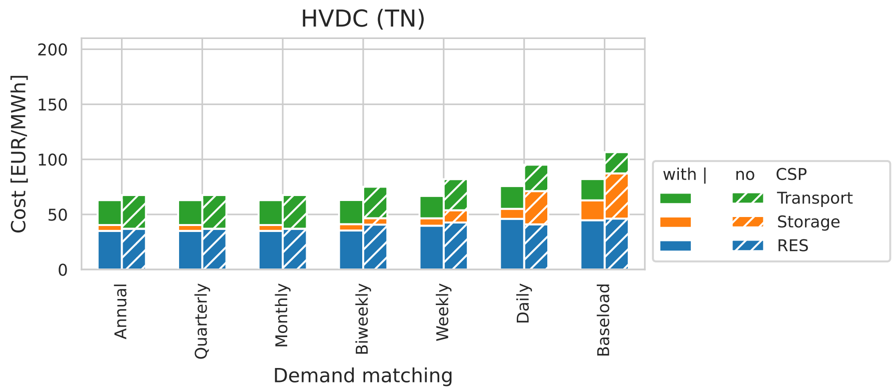

(b) HVDC from TN.

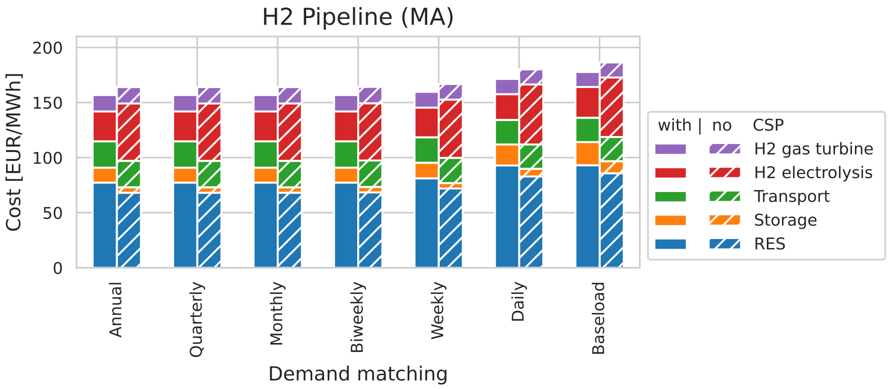

(c) H_2 Pipeline from MA.

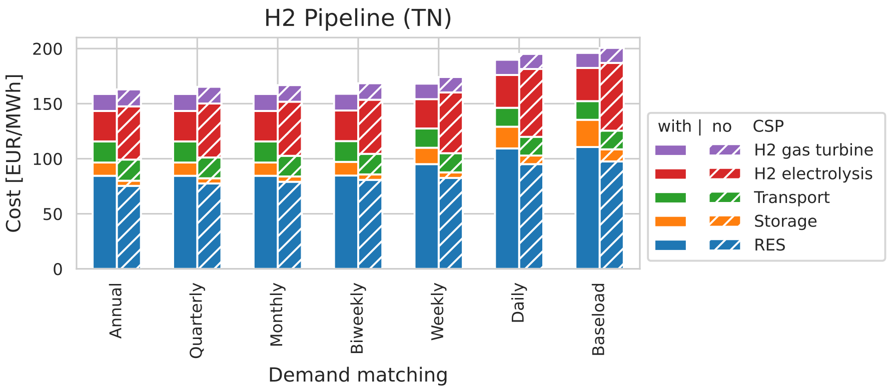

(d) H_2 Pipeline from TN.

Fig A. Cost compositions in EUR/MWh for all scenarios calculated in this study with and without CSP.

(a) HVDC from MA, (b) HVDC from TN, (c) H_2 pipeline from MA, (d) H_2 pipeline from TN. Extended and additional versions of Fig 7. Hatched bars indicate scenarios without CSP and TES, solid bars with both technologies.

S3B Appendix. Capacities for storage, transport and Hydrogen technologies

In Fig 6 the capacities for storage, transport, and H_2 technologies for ESCs from MA with CSP are shown. An extended version of this figure is shown below in Fig B for all flexibility scenarios, scenarios without CSP, and all scenarios from TN.

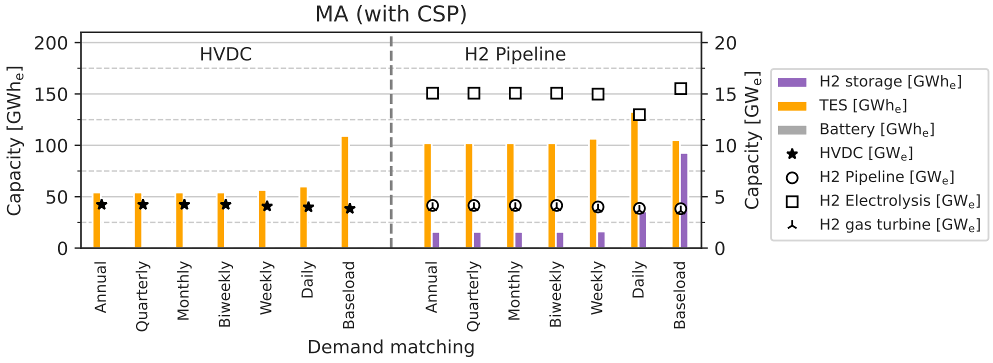

(a) ESCs from MA with CSP and TES.

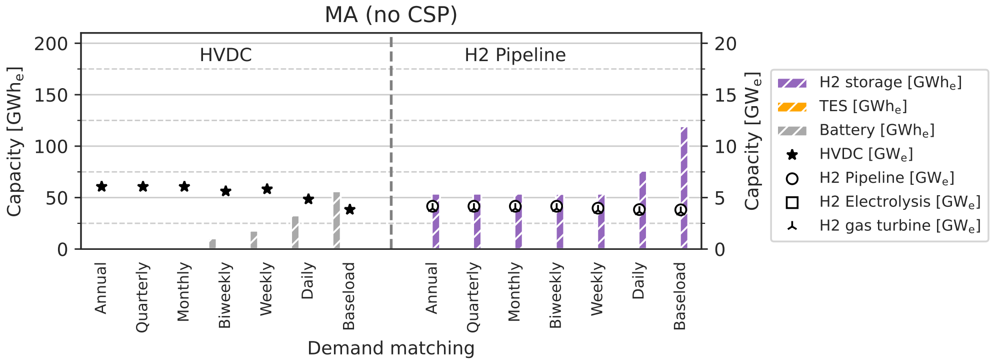

(b) ESCs from MA without CSP and TES.

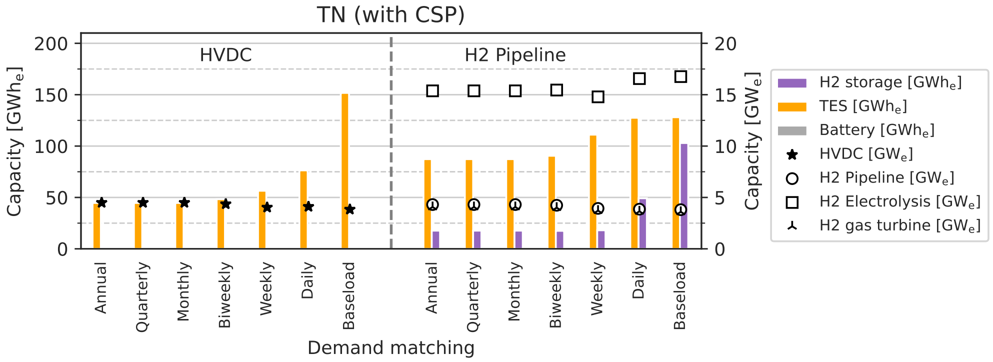

(c) ESCs from TN with CSP and TES.

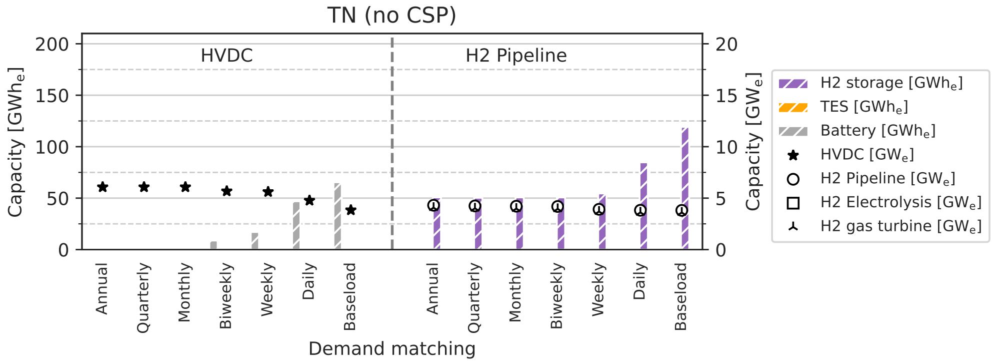

(d) ESCs from TN without CSP and TES.

Fig B. Capacities for storage, transport, and H_2 technologies.
(a) from MA with CSP, (b) from MA without CSP, (c) from TN with CSP, (d) from TN without CSP. Extended and additional versions of Fig 6. All capacities are reported in electricity equivalents, see text for details. Hatched bars indicate scenarios without CSP and TES, solid bars with both technologies.

S3C Appendix. Electricity mix

In Fig 4 and Fig 5 the capacities for storage, transport, and H_2 technologies for ESCs from MA with and without CSP are shown. An extended version of this figure is included below for all flexibility scenarios, scenarios without CSP, and all scenarios from TN.

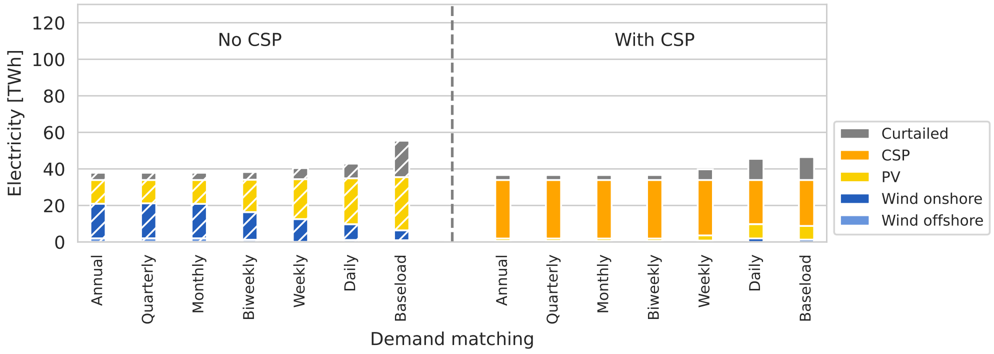

(a) HVDC from MA.

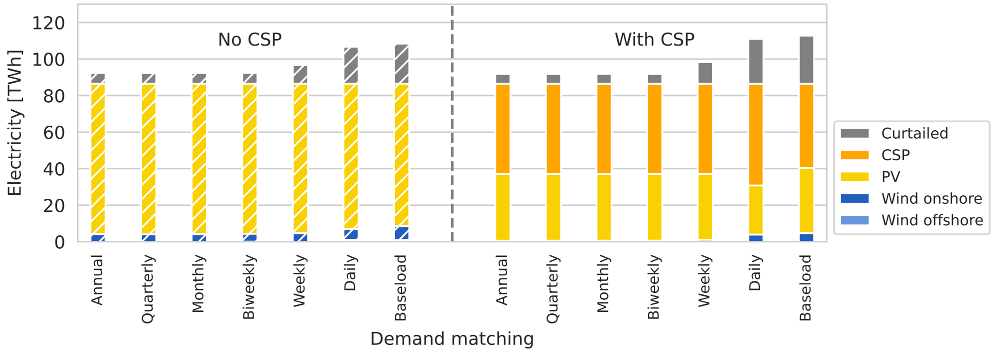

(b) H_2 pipeline from MA.

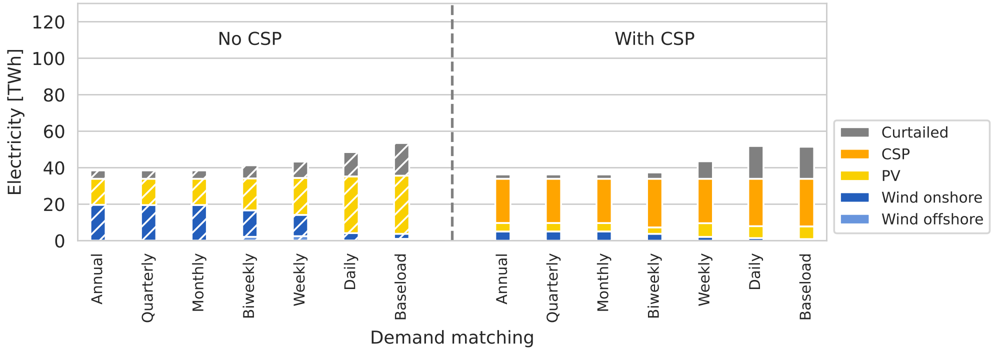

(c) HVDC from TN.

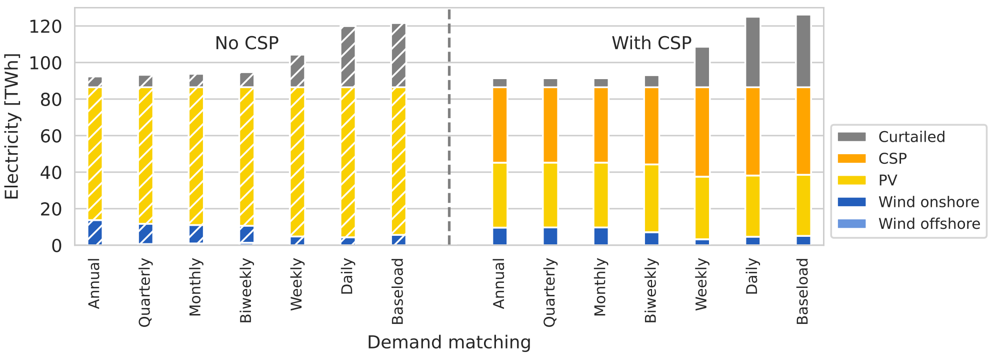

(d) H_2 pipeline from TN.

Fig C. Electricity generation mix for ESCs without and with CSP and TES technologies available to the model.
(a) for HVDC from MA, (b) for H_2 pipeline from MA, (c) for HVDC from TN, (d) for H_2 pipeline from TN. Extended and additional versions of Fig 4 and Fig 5. Hatched bars indicate scenarios without CSP and TES, solid bars with both technologies.
