# Supplementary material for: Flexing with lines or pipes: Techno-economic comparison of renewable electricity import options for European research facilities
Source: PLoS One. 2024 Feb 8;19(2):e0292892. doi: 10.1371/journal.pone.0292892 (PMC10852270; doi:10.1371/journal.pone.0292892)
Supplement: S4 Appendix — (PDF) [file pone.0292892.s004.pdf]

S4 Appendix. Regions and land availability analysis results

S4A Appendix. Morocco

The following administrative regions based on Database of Global Administrative Areas (GADM) [1] were included in the analysis for MA: Tanger - Tétouan, Gharb - Chrarda - Béni Hssen, Taza - Al Hoceima - Taounate, Rabat - Salé - Zemmour - Zaer, Grand Casablanca, Fès - Boulemane, Oriental, Chaouia - Ouardigha, Doukkala - Abda, Tadla - Azilal, Meknès - Tafilalet, Marrakech - Tensift - Al Haouz, Souss - Massa - Draâ.

Eligible land for individual technologies after land availability analysis is shown below in Fig D.

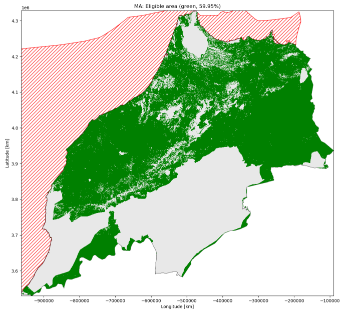

(a) Solar PV.

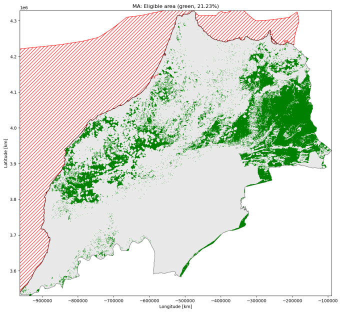

(b) Solar CSP.

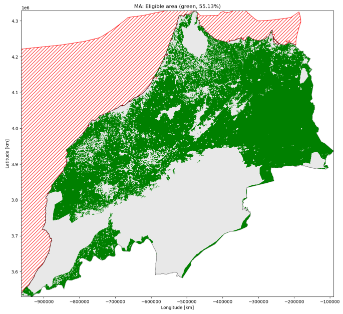

(c) Wind onshore.

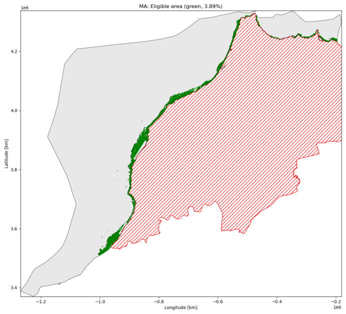

(d) Wind offshore.

Fig D. Areas in Tunisia considered eligible and considered for RES. (a) for solar PV, (b) for solar CSP, (c) for wind onshore, (d) for wind offshore.

S4B Appendix. Tunisia The following administrative regions based on GADM [1] were included in the analysis for TN: Bizerte, Ariana, Tunis, Manubah, Béja, Nabeul, Ben Arous (Tunis Sud), Jendouba, Zaghouan, Siliana, Le Kef, Sousse, Kairouan, Monastir, Mahdia, Kassérine, Sidi Bou Zid, Sfax, Gafsa, Tozeur, Gabès, Kebili, Médenine.

Eligible land for individual technologies after land availability analysis is shown below in Fig E.

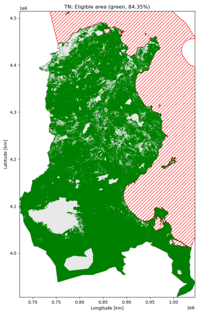

(a) Solar PV.

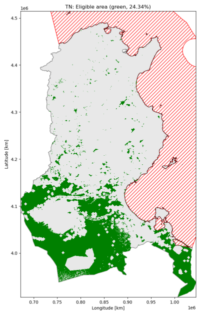

(b) Solar CSP.

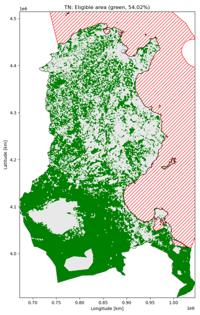

(c) Wind onshore.

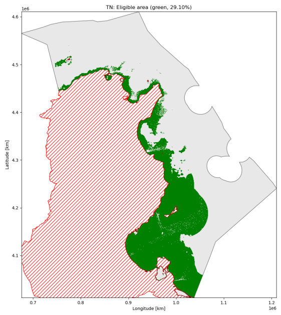

(d) Wind offshore.

Fig E. Areas in Tunisia considered eligible and considered for RES. (a) for solar PV, (b) for solar CSP, (c) for wind onshore, (d) for wind offshore.

References

1. Global Administrative Areas. GADM Version 3.6; 2018. Available from: https://gadm.org/download_world36.html.
