# Supplementary material for: Flexing with lines or pipes: Techno-economic comparison of renewable electricity import options for European research facilities
Source: PLoS One. 2024 Feb 8;19(2):e0292892. doi: 10.1371/journal.pone.0292892 (PMC10852270; doi:10.1371/journal.pone.0292892)
Supplement: Attachment — Submitted filename: response-to-reviewers.pdf [file pone.0292892.s005.pdf]

Individual responses

0 Formatting and color codes used in reponses 2

1 Academic Editor 3

2 Reviewer #1 4

2.1 Introduction 4

2.2 Method 5

2.3 Results and discussion 6

2.4 References 7

3 Reviewer #2 8

4 Reviewer #3 10

0 Formatting and color codes used in reponses

I use the following formatting and color coding for our response:

> *Comment:*
> Comments I received from the editor and reviewers are formatted like this.

> *Response:*
> My responses or actions I took based on the comments received are formatted like this.
>
> > 'Direct quotes from my manuscript are formatted like this.'

1 Academic Editor

Comment:
1. Please ensure that your manuscript meets PLOS ONE's style requirements, including those for file naming. The PLOS ONE style templates can be found at https://journals.plos.org/plosone/s/file?id=wjVg/PLOSOne_formatting_sample_main_body.pdf and https://journals.plos.org/plosone/s/file?id=ba62/PLOSOne_formatting_sample_title_authors_affiliations.pdf

Response:
The manuscript has been updated to meet the PLOS ONE style requirements.

Comment:
2. We note that Figure 2 in your submission contain [map/satellite] images which may be copyrighted. All PLOS content is published under the Creative Commons Attribution License (CC BY 4.0), which means that the manuscript, images, and Supporting Information files will be freely available online, and any third party is permitted to access, download, copy, distribute, and use these materials in any way, even commercially, with proper attribution. For these reasons, we cannot publish previously copyrighted maps or satellite images created using proprietary data, such as Google software (Google Maps, Street View, and Earth). For more information, see our copyright guidelines: http://journals.plos.org/plosone/s/licenses-and-copyright.
We require you to either (a) present written permission from the copyright holder to publish these figures specifically under the CC BY 4.0 license, or (b) remove the figures from your submission: [. . .]

Response:
No additional permissions are necessary for this figure. The map shown in Figure 2 was created by myself. It is based on the two datasets also cited in the figure description, NaturalEarth and GEBCO. Both datasets are released into the public domain and therefore do not require any written permission for use.

2 Reviewer #1

The manuscript entitled 'Flexing with lines or pipes: Techno-economic comparison of renewable electricity import options for European research facilities' conducts a techno-economic analysis of long-distance electricity imports from variable renewable energy sources. It also compares direct imports of electricity by High Voltage Direct Current transmission line with indirect imports by hydrogen pipelines. It is commendable that the authors included a wide range of perspectives and factors in the study. Meanwhile, extensive revision is necessary to make it suitable for publication. Here are some further remarks in more detail.

2.1 Introduction

> *Comment:*
> The background of the study is well presented. However, some literature needs to be reviewed to aid identify what has been done, what is being done currently, and what needs to be done in relation to this research area.

> *Response:*
> Thank you for taking the time to review the paper and for your positive feedback! Your comments and suggestions are highly appreciated.

> *Comment:*
> Provide more information in the introduction section, on how renewable energy sources contribute to clean energy. In this sense, the following materials may be useful:
>
> - https://doi.org/10.1016/j.biteb.2022.101167
> - https://doi.org/10.1016/j.nexus.2022.100157

> *Response:*
> The revised version contains a short note on how renewables can help to reduce scope 2 emissions of research institutions. A general discussion of their contribution and role would go beyond the scope of this paper.

> *Comment:*
> The novelty of this paper should be presented concisely and clearly.

Response:
The introduction has been revised to highlight the novelty of the paper.

2.2 Method

Comment:
* Transparency and replicability are two of the most crucial characteristics of good modern science, but the findings of this paper - as they currently stand, cannot be replicated because the authors have not provided enough information about their modelling techniques, input data, and underlying assumptions. For instance, what were the economic criteria used to appraise the proposed power project? Is it lifecycle cost, annualised cost, or levelised cost of energy? Furthermore, the technical criteria for appraising the proposed project should be detailed in the paper. Both technical and economic models used should be expressed using necessary mathematical equations. On this note, I recommend to you these papers for various technical and economic metrics for analysing the performances of renewable energy systems.

(i) https://doi.org/10.3390/pr8111381

(ii) https://doi.org/10.1007/s41660-022-00223-9

Response:
I expanded the methods section with key aspects of the methodology and discounting method used from Hampp et al. (2023), which describes the methodology used here in greater detail. Specifics on the techno-economic assumptions have been moved from the open data repository to the supplementary materials.
The different scenarios were evaluated based on their Levelised Cost of Energy (LCoE), generation and storage capacities, annual electricity generation volumes, and the cost compositions, i.e. a view on the cost drivers of import costs within the specific scenarios.

Comment:
Detailed capacities/ratings of components (i.e. battery, CSP, PV, wind turbines, TES, inverter, etc.) and their models with appropriate justifications for their choice should be specified.

Response:
Detailed capacities are shown in the supplementary information S1 Figures B and C. I've added the details on the technology assumptions as Table A in S1 Table; the details were

previously only contained in the Zenodo repository. In addition the following sentence was added to the text:

> ‘ Costs are assumed for large-scale facilities and scale linearly with capacity, no additional scaling exponent is applied. ’

Comment:
An overview of the software tool (PyPSA and atlite) used in this research should be presented.

Response:
I have added additional information on PyPSA and atlite. For a more detailed description of both software packages the reader can refer to the cited publications. A more detailed description of their underlying functionings are beyond the scope of this paper.

2.3 Results and discussion

Comment:
The authors have ample room to present and analyse the outcomes of their model, in relation to the larger body of literature. That is, the results and discussion section should be strengthened in the context of the related literature.

Response:
Thank you for the suggestion, I have expanded the discussion section accordingly.

Comment:
Fig. 3 is too low-resolution and blurry. The quality of the figure should be improved.

Response:
Thank you for your keen eye. I have increased the size of the figure and ensured that it is now a vector graphic to avoid any low-resolution issues.

2.4 References

Comment:
Citation is improperly done. References are not arranged in an orderly manner. For instance, the first paper cited in the introduction section has a reference number [29]. The authors are advised to follow the journal's author guidelines for revision.

Response:
Journal formatting requirements have been adapted with the revision.

3 Reviewer #2

Comment:
Overall the paper is of good quality. However, before approval needs some changes.

Response:
Thank you for taking the time to review the paper and for your positive feedback! Your comments and suggestions are highly appreciated.

Comment:
1. Abstract should be made more sound by including objective, policy implications, data period etc.

Response:
The abstract has been revised.

Comment:
2. Introduction should be made more smooth by removing redundant lines.

Response:
The introduction has been revised.

Comment:
3. Literature gap and contribution should also be highlighted at the end of introduction.

Response:
The introduction has been revised to highlight the gap the paper addresses in relation to existing literature.

Comment:
4. Methods must also include hypothesis which are tested in the analysis.

Response:
Given the explorative nature of the study, I do not consider the formulation of a hypothesis and testing for it a suitable approach.

Comment:
5. Results must also discuss in the light of past empirical and theoretical findings

Response:
As this study is explorative, no relevant empirical results to compare against exist. As for other theoretical findings, I am not aware of studies with comparable design and scope which the study could be directly compared against. The only relevant other study with comparable relevance I'm currently aware of is included in the discussion section (Walter et al. (2023)).

Comment:
6. Conclusion must also include policy suggestions.

Response:
Thank you for the suggestion. Given the general nature of the results of this study, I believe it is inappropriate to formulate concrete policy recommendations.

4 Reviewer #3

Comment:
Thank you for the opportunity to peer-review this paper. The paper uses a techno-economic analysis to compare the feasibility of direct imports of electricity by High-Voltage Direct Current transmission line with indirect imports by hydrogen pipeline for long-distance electricity imports from North Africa to Central Europe. The benefits and trade-offs of demand-side flexibility and Concentrated Solar Power with Thermal Energy Storage on the system storage requirements are also investigated. The following observations have been raised;

Response:
Thank you for taking the time to review the paper! Your comments and suggestions are very welcome.

Comment:
1. The word 'Longdistance' should be separated in the abstract.

Response:
The revised abstract now contains the compound word 'long-distance'.

Comment:
2. The abstract is not informative, it should be improved, and numerical results clearly stated.

Response:
The informative results of the study are the qualitative differences between the scenarios: hydrogen vs. electricity and the effect of demand flexibility. For these results, the quantitative numbers are provided. To keep the abstract short, the interested reader can find more and detailed numbers in the manuscript.

Comment:
3. In the introduction, instead of focusing on novelty and originality, the literature review should be enhanced and updated. It is important to explain how the proposed approach adds value compared to existing research. Additionally, the study's relevance and significance should be established by identifying the limitations of previous publications that this research aims to address. Please refer to: https://doi.org/10.3390/math11

051213 https://doi.org/10.1016/j.desal.2023.116376 https://doi.org/10.1016/j.jhydrol.2020.124974

Response:
I have revised the introduction accordingly.

Comment:
4. Study area is poorly described, rather it is unclear. Why are these stations selected? What are their particularities? More details is necessary.

Response:
A short explanation of the selection criteria for the starting points of the import routes has been added. For the applied methodology, the exact starting point is of no major relevance, only each route's distance influences the results.

Comment:
5. A lot of points have been raised in the first paragraph of the Introduction but not many citations to support them. No reference was given for the 'Morocco-Uk Power Project' mentioned.

Response:
Thank you for pointing out the missing citation. I've added a number of references were added to the introduction for aspects which might not be considered general knowledge outside the modeling field and the missing citation to Xlinks.

Comment:
6. Provide a more detailed explanation of the research methodology and data analysis techniques used in the paper.

Response:
Section '2 Method' was revised to state the key aspects of the methodology used. For a more detailed explanation of the methodology, the reader is referred to Hampp et al. (2023), previously previously published in PLOS ONE, which contains a detailed explanation of the methodology.

Comment:
7. Include a more comprehensive literature review to provide a better context for the research question and its significance.

Response:
A short literature review and relevant references were added.

Comment:
8. In the Methods section, 2.1 General, the first paragraph should be rephrased to make the point clearer.

Response:
The paragraph was rephrased.

Comment:
9. In text citations of the figures were written as ‘Figure’ in full instead of ‘Fig’ as shown in the guidelines.

Response:
Journal formatting requirements have been adapted with the revision.

Comment:
10. In subsection 2.4, Table 1, the second column, the first letter of ‘investment’ should be capitalized.

Response:
Adapted.

Comment:
11. The author should provide a more detailed discussion of the implications and potential impact of the research findings.

Response:
Thank you for your suggestion, I have expanded the discussion section accordingly.

Comment:
12. Collaborate with experts in related fields to provide a more interdisciplinary perspective on the research question.

Response:
An interdisciplinary investigation of the results is a good idea for a follow-up project. For this paper, the decision was intentionally made to limit the disciplinary scope, as the purpose of the paper is to discuss the effects of demand-side flexibility in the specific context from a techno-economic modeling perspective.
